# Otto Cars: reacting to antimicrobial resistance

**DOI:** 10.2471/BLT.19.030619

**Published:** 2019-06-01

## Abstract

Otto Cars talks to Gary Humphreys about the lack of progress on antimicrobial resistance (AMR) and the urgent need for comprehensive, cross-sectoral action.

Q: You started Action on Antibiotic Resistance (ReAct) over a decade ago. Looking back, did you expect it to be so hard to make progress on AMR?

A: Perhaps, but I think we went into this with our eyes open. You have to remember that one of the reasons we set up ReAct was precisely to address the lack of progress. In fact, my colleagues and I invited WHO to discuss the issue, and in particular why there had been so little reaction to the 2001 *WHO Global Strategy for Containment of Antimicrobial Resistance*. It was after that consultation that we decided to create an organization that would drive advocacy, but also support the collection and generation of evidence and coordinated action. Having said that, it’s clear that progress really has been very slow. The understanding of the dimensions of AMR has increased at the professional level, but to a lesser degree within governments and civil society. Things changed after the launch of the *Global action plan on antimicrobial resistance* in 2015, but we are still far from mounting a serious response to what is a very serious problem.

Q: Why do you think there has been so little progress?

A: One reason is failure to communicate effectively with policy makers and donors. The AMR story is a difficult story to tell because it is not one disease; it is something that undermines the treatment of many diseases and therefore health systems. Also, it is not something that you can identify easily, such as malaria or HIV. The sheer complexity of the issue is another challenge; we have to move the discussion away from genes and bacteria and focus upon the transformation of failed systems.

Q: What do you mean by that?

A: We have tended to look at AMR as a narrow technical issue that can be fixed with some research funding and new antibiotics. However, to push back against AMR we need robust health systems. Tackling AMR starts with infection prevention, the effective use of existing vaccines, and ensuring safe water and sanitation. Any effort to move towards universal health coverage must take into account the many ways in which antimicrobial resistance will impact health systems as countries expand coverage and access. We also need to look beyond health, because AMR is an agricultural sector issue too, as well as an ecological issue. The tripartite agencies: WHO, the Food and Agriculture Organization of the United Nations and the World Organisation for Animal Health have a critical role to play, but collaboration needs also to be expanded to include other agencies, such as the United Nations Environment Programme, the United Nations Children's Fund and the United Nations Development Programme.

“We also need to look beyond health, because AMR is an agricultural sector issue too, as well as an ecological issue.”

Q: Speaking as an epidemiologist, how would you characterize the antimicrobial resistance epidemic?

A: We are talking about a slow pandemic. Of immediate concern is the spread of the most resistant gram-negative bacteria, including Acinetobacter, *Escherichia coli* and *Klebsiella pneumoniae*. These bacteria are increasingly resistant to most antibiotics and cause infections ranging from pneumonia, bloodstream infections, wound or surgical site infections, and meningitis. This kind of resistance is something we have observed for three decades or more and the death toll is accelerating.

Q: Some commentators have suggested that the development of transferable colistin resistance means that we have already entered the post-antibiotic era. Do you agree with that assessment?

A: The discovery of the mobilized colistin resistance gene is clearly a matter of concern, since bacteria carrying such genes are often resistant also to most other antibiotics. But, regardless of that development, the post-antibiotic era has already arrived for many people, especially those in low-income countries or people trapped in humanitarian emergencies who cannot get access to effective medicines. One of the most striking examples is Malawi where according to a recent study, Klebsiella resistance jumped from 12% to 90% of infections between 2003 and 2016. In East Mosel, in the Syrian Arab Republic, Médecins Sans Frontières’ staff recently reported that almost 40% of the patients admitted to their centres had multi-resistant infections. Of course, AMR is also present in high-income countries. Even here in Sweden we must sometimes rely on intravenous antibiotics for simple urinary tract infections because none of the oral medicines work. So treatment practices are already changing, with negative consequences for patients, while also driving up health care costs. We are clearly on the brink of a major crisis.

Q: What can we do about it?

A: The ultimate responsibility falls upon governments, but the whole of society needs to act now. We absolutely know as much as we need to about the dynamics of resistance development, and its consequences in different parts of the world - not least in terms of the economic damage it does. AMR is the result of multiple system failures and can only be managed by balancing innovation, access and conservation. So we have to act now on the basis of the data we have, while also acknowledging that we don't yet have a fully developed epidemiological picture.

Q: Does that mean that you do not support the idea of developing the global resistance surveillance system?

A: Efforts to improve surveillance are of course critical and I recognize the importance of the Global Antimicrobial Resistance Surveillance System (GLASS), which started well, but is still in an early stage of development. However, there are limits to what such systems can do in the near term and we have to recognize the costs of delaying action while waiting for such a system to be fully built.

Q: What are those limits, in your view?

A: Well, simply put, the picture is necessarily incomplete. And that is a reflection of the capacity constraints faced by many countries. Surveillance systems require a lot of resources. Numerous samples have to be collected and transported to laboratories that have the required quality and capacity. Most European countries have well-developed surveillance systems that have been built up over many years. But in low-income countries, data are still scarce and there is a dramatic lack of data on antibiotic susceptibility in common bacteria, which are needed to guide empiric therapy. So, we need to think about approaches that can quickly generate high-quality data in hospitals, health facilities and communities.

“The post-antibiotic era has already arrived for many people, especially those in low-income countries.”

Q: What kind of approaches do you have in mind?

A: I believe we need to focus efforts on conducting point prevalence studies. Such studies can be done by researchers or health ministries at relatively low cost, and without causing major infrastructural challenges. Given the difficulty that many low- and middle-income countries have in mobilizing resources for even basic AMR-related activities, it may be necessary to use external catalytic funding to galvanize activity in this area. Information can be captured regarding specific bacterial species, resistance patterns, antibiotic access and use, guideline adherence or hospital acquired infections. 

Q: There has been a good deal of discussion regarding the lack of new antimicrobials in the research and development pipeline. What can be done about this?

A: The whole research and development ecosystem needs to be rebuilt and the public sector needs to take leadership. Ensuring sustainable access for antibiotics calls for a new paradigm – an end-to-end approach that addresses the entire lifecycle of a drug, from solving the scientific challenges to ensuring equitable access. This will require policy interventions at every stage of the pharmaceutical value chain, as well as the coordination of such efforts. While there is no single “correct” formula, I believe that new incentives to develop antibiotics must separate the cost of research and development from the volume of sales and end prices of antibiotics.

Q: In a recent WHO Bulletin interview, Mirfin Mpundu, head of ReAct Africa, pointed out that of the 25 African countries that have national action plans, only three or four are actually implementing them. What can be done to get governments to do more?

A: The problem of inaction is not limited to Africa. There are national action plans in over 100 countries, but only around a fifth of these action plans are properly funded. I support the idea of mobilizing external catalytic funding to galvanize the implementation of national action plans. I also believe we need to open a dialogue with ministers of finance and development, making it clear that AMR is a development issue and the costs of not addressing it will be high, both in terms of human health and economic growth. Beyond that, I believe we need to consider some form of binding agreements. We need to set targets that measure progress and hold governments and professionals accountable. The Interagency Coordination Group recommends the establishment of a global leadership group and an independent panel on evidence for action. These can encourage public and private financing, support the monitoring of progress, and provide robust and authoritative assessments of the science across all sectors. They can also help generate the political will required and promote new global collaboration. The fact is that we have failed to look upon effective antimicrobials as a shared global resource. The time has come to do something about that.

